# Concordance between self-reported pre-pregnancy body mass index (BMI) and BMI measured at the first prenatal study contact

**DOI:** 10.1186/s12884-016-0983-z

**Published:** 2016-07-26

**Authors:** Barnabas K. Natamba, Sixto E. Sanchez, Bizu Gelaye, Michelle A. Williams

**Affiliations:** 1 Department of Epidemiology, Harvard TH Chan School of Public Health, 677 Huntington Avenue, Kresge Room 500, Boston, MA 02115 USA; 2 Universidad Peruana de Ciencias Aplicadas, Lima, Peru; 3 Asociacion Civil Proyectos en Salud (PROESA), Lima, Peru

**Keywords:** Self-reported pre-pregnancy weight, height and BMI, Weight, height, and BMI measured early during pregnancy, Concordance, Peru, South America, Low- and middle–income country

## Abstract

**Background:**

The 2009 Institute of Medicine (IOM) gestational weight recommendations are tailored to women’s pre-pregnancy body mass index (BMI). Limited evidence exists on methods for estimating women’s pre-pregnancy BMI, particularly for women living in low and middle income countries. Using data from collected among Peruvian pregnant women, we compared the concordance between self-reported pre-pregnancy BMI with BMI measured at the earliest prenatal study visit.

**Methods:**

Data were from the Pregnancy Outcomes Maternal and Infant Study (PrOMIS), a cohort of pregnant women at the Instituto Nacional Materno Perinatal (INMP) in Lima, Peru. 2605 women aged 18 to 49 years (mean ± SD gestational age = 10.9 ± 3.3 weeks) were included in the study. Self-reported pre-pregnancy weight and height and measured weight and height were collected at the first prenatal study contact. We assessed the concordance between measured and self-reported BMI; and, the agreement among indicators of nutritional status obtained using measured and self-reported BMI.

**Results:**

On average, weight measured at the first prenatal study visit was 0.27 kg higher than self-reported pre-pregnancy weight (*p* < 0.05); and, measured height was 0.02 m lower than self-reported pre-pregnancy height (*p* < 0.001). Correspondingly, measured BMI was 0.71 kg/m^2^ higher than self-reported BMI (*p* < 0.001). Scatter and Bland-Altman plots indicated strong concordance between measured and self-reported BMI. The proportion of women in the normal BMI category tended to be higher when using self-reported BMI (59.6 %) than when using measured BMI (50.4 %). Conversely, the proportion of women in the overweight or obese BMI categories tended to be lower when using self-reported BMI (38.2 %) than when using measured BMI (47.7 %).

**Conclusion:**

Self-reported pre-pregnancy BMI was strongly correlated with BMI measured at the first prenatal study contact. The findings potentially suggest that, in this context, there is minimal change between pre-pregnancy BMI and BMI measured at the first prenatal study contact; or, that women in this study just recalled their most recent measured anthropometrics (including values obtained during the index pregnancy but before enrollment in the PrOMIS study).

**Electronic supplementary material:**

The online version of this article (doi:10.1186/s12884-016-0983-z) contains supplementary material, which is available to authorized users.

## Background

While there is no global consensus on gestational weight gain (GWG) recommendations, many countries base their GWG guidelines or policies on women’s pre-pregnancy nutritional status [1]. For instance, the 1990 and the 2009 Institute of Medicine (IOM) GWG recommendations in the US are based on women’s pre-pregnancy body mass index (BMI: calculated as weight in kg divided by the square of height in meters) category [2, 3]. To highlight, women entering pregnancy with normal BMI (i.e. 18.5–24.9 kg/m^2^ according to the World Health Organization classifications) are recommended to gain, on average, 0.5 kg per week during the second and third trimesters. There is evidence, for example, that approximately 40–60 % of US women gain weight in excess of the 2009 IOM recommendations while 15–20 % gain less than what is recommended [4–6]. Gaining weight within IOM recommendations of each BMI category prevents adverse maternal and neonatal outcomes associated with excessive or inadequate GWG. For pregnant women, excessive GWG has been associated with several adverse health outcomes including pre-eclampsia, caesarian delivery, gestational diabetes, and postpartum weight retention [7–12]. For neonates, excessive weight gain during pregnancy is associated with being born large for gestational age, low 5 min Apgar scores, seizures, and childhood obesity [4, 13, 14]. Inadequate GWG, on the other hand, has been linked to small for gestational age neonates and preterm birth [4, 10, 15].

To comply with IOM or other nationally approved GWG recommendations, it is recommended to use pre-pregnancy BMI obtained from weight and height measured at the most recent pre-conception visit [2]. If available, measured pre-pregnancy anthropometrics (weight and height) would enable midwives and obstetricians diagnose women’s pre-pregnancy nutritional status (BMI). Measured maternal pre-pregnancy BMI, however, is difficult to obtain as a majority of pregnancies are not planned; and, even among women with planned pregnancies, the majority do not seek pre-conceptional care. In the US, for example, it has been reported that 50 % of all pregnancies are not planned [16]. In low and middle income countries (LMICs), such as Peru, the proportions of women with unplanned pregnancies or who do not seek pre-conceptional care are unknown; and, could be more prevalent than the reported values for high income settings. As such, in most contexts, measured pre-pregnancy anthropometrics are rarely available for most women entering pregnancy. In their absence, clinicians and researchers can rely on self-reported pre-pregnancy anthropometrics or anthropometrics measured at the earliest prenatal contact to obtain a reliable estimate of pre-pregnancy BMI and to facilitate counseling on GWG [17, 18]. Indeed, use of self-reported weight and height for estimation of pre-pregnancy BMI [19, 20] or in other contexts [21, 22] is a common feature in nutritional assessment. Studies conducted mainly in high income countries have demonstrated the extent to which self-reported pre-pregnancy BMI compares to its proxies including imputed values of pre-pregnancy BMI [19] or BMI measured early during pregnancy [19, 20]. Limited data exist on the level of agreement between self-reported pre-pregnancy BMI and BMI measured at the earliest prenatal contact among women living in LMICs.

Therefore, using data collected among Peruvian pregnant women, we studied the concordance between BMI obtained from self-reported pre-pregnancy weight and height and BMI from measured at the earliest prenatal study contact (mean ± SD gestational age: 10.9 ± 3.3 weeks).

## Methods

Data presented in this study were analyzed from the Pregnancy Outcomes, Maternal and Infant Study (PROMIS) cohort, an ongoing prospective study of pregnant women enrolled in prenatal clinics at the Instituto Nacional Materno Perinatal (INMP) in Lima, Peru. The INMP is the main referral hospital for maternal and perinatal care in Peru. Methodological details of the PrOMIS study cohort have been previously published [23, 24]. Briefly, women were eligible for inclusion in this study if they initiated prenatal care prior to 18 weeks of gestation. Between February 2012 and March 2014, 3162 women who had their first prenatal care visit at INMP met the 18 weeks of gestation eligibility criteria. Women were excluded from the study if they were younger than 18 years, did not speak Spanish or were more than 18 weeks of gestation.

### Sociodemographic information

Trained personnel used structured questionnaires to interview study participants in a private setting. Sociodemographic and lifestyle information, medical and reproductive history were collected. Participants’ age was categorized as follows: 18–19, 20–29, 30–34, 35 year or older. Other socio-demographic variables included: ethnicity (Mestizo vs. other); marital status (married or living with a partner vs. other); educational attainment (6 years or lower, 7–12 years, and greater than 12 years); employment status during pregnancy (employed vs. not employed); parity (nulliparous vs. multiparous); smoking status before this pregnancy (smoked vs. never smoked); and, smoking status in the index pregnancy (smoking vs. not smoking).

### Anthropometrics

During interviews, participants were asked to report their pre-pregnancy body weight and height. At the first prenatal study visit, and after interviews were completed, trained research nurses measured participants’ anthropometrics (weight and height) as per standard anthropometric procedures [25]. Briefly, body weight (in kg to the nearest 0.1 kg) was measured using a reliable digital weighing scale (Soehnle Solar Fir , Leifheit AG, Leifheitstrabe 1, 56377, Nassau/Germany) while participants were wearing light clothing and no shoes. Body height (in cm to the nearest 0.1 cm) was measured using portable Shorr-type wall mounted height board with a measuring slide and a heel plate. The position of the head was standardized by asking participants to stand straight without shoes and with the heels together. Height and weight were measured twice without delay between measurements and a third measurement was taken when the first two measurements differed by ≥0.5 kg or ≥1.0 cm.

### Statistical analyses

Frequency distributions (counts and percentages) and measures of central tendency (mean, median, standard deviations) of sociodemographic and lifestyle characteristics of participants were examined first. Self-reported pre-pregnancy BMI and BMI from weight and height measured at the first prenatal study contact were calculated as weight (in kilograms) divided by height (in meters) squared. BMI was categorized based on the WHO guidelines in four categories (underweight: <18.5 kg/m^2^, normal: 18.5–24.9 kg/m^2^, overweight: 25.0–29.9 kg/m^2^, and obese >30 kg/m^2^). Histograms, normal quantile plots, skewness and kurtosis normality tests were used to assess the distributional properties of self-reported and measured height, weight and BMI. Measures of height, weight and BMI were considered to approximate a normal distribution if absolute values of skewness we less than one (<1) [26]. As perfect normal distributions are expected to have kurtosis values of three (3) [26], we took kurtosis values between 3 and 5 to approximate normality. We used paired t tests to assess statistically significant differences between measured and self-reported values. Concordance between measured and self-reported prevalence of underweight, normal, overweight and obese nutritional categories was assessed using Cohen’s weighted kappa statistic [27]. In addition, we used Scatter and Bland-Altman plots [28] to examine individual concordance between self-reported pre-pregnancy and measured BMI during the interview. The Y-axis of the Bland-Altman plots was based on differences between self-reported BMI and measured BMI. The X-axis was the mean of self-reported pre-pregnancy BMI and mean of measured BMI. Limits of agreement were calculated as the mean difference (MD) ± 1.96*standard deviations (SD). All analyses were performed using STATA software (version 14, StataCorp, College Station, TX, USA). The level of statistical significance was set at *p* < 0.05 and all tests were two sided.

## Results

At the time of writing this report, 3162 pregnant women had been recruited into the PrOMIS cohort study. Of these, 2605 participants (82.4 % of the original sample) with complete information about self-reported pre-pregnancy weight and height and weight and height measured at the first prenatal study contact were included in this analysis. Sociodemographic and lifestyle characteristics of the total sample are summarized in Table 1. In addition, in Additional file 1: Table S1, we summarize differences in sociodemographic and lifestyle characteristics of pregnant women that provided self-reported pre-pregnancy anthropometrics versus those that did not report such measures. Overall, participants’ (mean ± SD) gestational age at recruitment was 10.9 ± 3.3 weeks; and, participants were 28.0 ± 6.2 years old. Further, majority (80.7 %) of participants were married or living with a partner, 50.7 % were nulliparous and 96.1 % had at least 7 years of education. A substantial proportion of participants (20.9 %) in this study reported smoking before the index pregnancy and 3.9 % reported smoking during the index pregnancy. There were differences and similarities between those who provided self-reported pre-pregnancy anthropometrics and those who did not (Additional file 1: Table S1). For instance, the group that provided self-reported BMI was more likely to be of a mixed (Mestizo) race, to be nulliparous and to report seven or more years of education. Women, who did not report pre-pregnancy BMI, were about a year older and more likely to be married or living with a partner. However, the two groups did not differ in terms of their employment status nor did they differ in terms of whether they had a planned or unplanned pregnancy.

**Table 1:** Socio-demographic and lifestyle characteristics of participants in the PrOMIS cohort

Variable	Mean ± SD^a^ or n (%)^b^
Gestational age at recruitment	10.9 ± 3.3 weeks
Maternal age	28.0 ± 6.2 years
Age category	
18–20	129 (5.0 %)
20–29	1488 (57.1 %)
30–34	544 (20.9 %)
≥ 35	444 (17.0 %)
Ethnicity	
Mestizo (Mixed race ancestry)	1980 (76.1 %)
Other	621 (23.9 %)
Marital status	
Married or living with partner	2095 (80.7 %)
Other	500 (19.3 %)
Parity	
Nulliparous	1316 (50.7 %)
Multiparous	1282 (49.4 %)
Education level (years of education)	
≤ 6	101 (3.9 %)
7–12	1394 (53.7 %)
> 12	1103 (42.4 %)
Employment status	
Employed	1200 (46.1 %)
Not employed	1404 (53.9 %)
This pregnancy was planned	
Planned	1097 (42.3 %)
Not planned	1498 (57.7 %)
Smoked before this pregnancy	
Yes	543 (20.9 %)
No	2055 (79.0 %)
Currently smoking in this pregnancy	
Yes	101 (3.9 %)
No	2495 (96.1 %)

^a^ *SD* standard deviation

^b^Due to missing data, percentages may not add up to 100 %

**Fig. 1:**
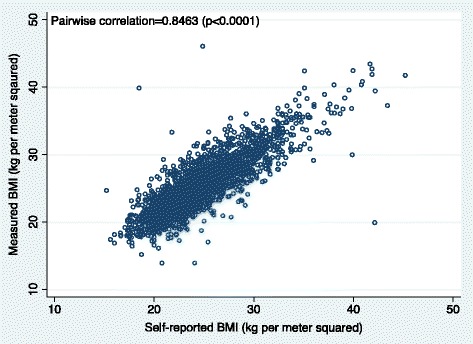
Scatterplot of BMI measured at the first prenatal study visit versus self-reported pre-pregnancy BMI

**Fig. 2:**
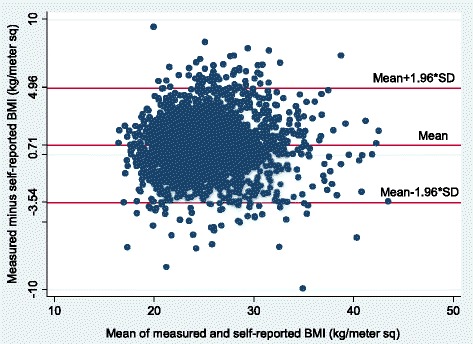
Bland-Altman plot for the difference in self-reported and measured BMI (with 95 % limits of agreement) against the mean of self-reported and measured BMI

Table 2 shows point estimates, differences, and statistical significance of the differences obtained from measured and self-reported pre-pregnancy anthropometrics. Examination of skewness (range: 0.13–1.00) and kurtosis (range: 3.04–4.76) measures, histograms and normal quantile plots indicated that self-reported and measured height, weight and BMI approximated normal distributions (Table 2). Weight measured at the first prenatal study contact was 0.27 kg significantly higher than self-reported pre-pregnancy weight (59.2 vs. 58.9 kg; *p* < 0.05) and measured height was 0.02 m significantly lower than self-reported pre-pregnancy height (1.53 vs. 1.55 m; *p* < 0.001). Correspondingly, BMI measured at the first prenatal study contact was 0.71 kg/m^2^ higher than self-reported pre-pregnancy BMI (25.4 vs. 24.7 kg/m^2^; *p* < 0.001). Scatter (Fig. 1; Pearson’s correlation co-efficient = 0.846, *p* < 0.0001) and Bland-Altman plots (Fig. 2) indicated strong concordance between measured and self-reported BMI. Further, Fig. 2 shows that, for most participants, differences between measured and self-reported BMI were within 1.96 SD of the overall mean difference and that this pattern was maintained across the mean of self-reported and measured BMI.

**Table 2:** Mean difference comparisons between measured and self-reported anthropometrics for pregnant women in the PrOMIS cohort (*N* = 2605)

Measure	Measured			Self-reported			Mean difference (SD)
	All	Skewness	Kurtosis	All	Skewness	Kurtosis	All
Weight (kg)	59.21 ± 10.05	0.87	4.26	58.94 ± 9.89	0.96	4.60	−0.27 ± 4.54*
Height (m)	1.53 ± 0.05	0.13	3.04	1.55 ± 0.06	0.19	3.08	+0.02 ± 0.03**
BMI (kg/m^2^)	25.35 ± 3.97	0.92	4.46	24.65 ± 3.87	1.00	4.76	−0.71 ± 2.17**

*p < 0.05

**p < 0.001

**Fig. 3:**
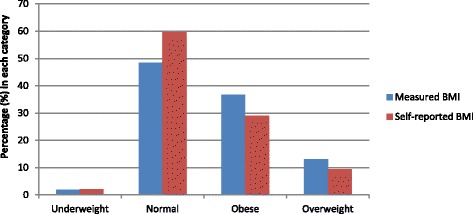
Percentage participants in the WHO BMI category by whether their BMI was measured or self-reported

The proportion of participants appropriately classified by self-reported BMI in each of the four categories of measured BMI (as per WHO classifications) were 41.7 % (Underweight category), 89.9 % (Normal weight category), 58 % (Overweight category) and 62.3 % (Obese category). The overall observed agreement was 74.5 % with a weighted kappa statistic of 0.73 (Table 3). Also, it can be observed in Fig. 3 that the proportion of women in the normal BMI category tended to be higher when using self-reported BMI (59.9 %) than when using measured BMI (50.4 %). Conversely, the proportion of women in the overweight or obese BMI categories tended to be lower when using self-reported BMI (38.2 %) than when using measured BMI (47.7 %) (Fig. 3).

**Table 3:** Statistical agreement between measured and self-reported BMI (kg/m^2^) (*N* = 2605)

	Measured BMI				
Self-reported BMI	Underweight	Normal	Overweight	Obese	
Underweight	20 (41.7 %)	32 (2.4 %)	0 (0.0 %)	1 (0.3 %)	Overall observed agreement =74.5 %; Weighted kappa statistic = 0.73
Normal	27 (56.3 %)	1,175 (89.9 %)	343 (36.0 %)	10 (3.4 %)	
Overweight	1 (2.0 %)	99 (7.6 %)	553 (58.0 %)	101 (34.0 %)	
Obese	0 (0.0 %)	1 (0.1 %)	57 (6.0 %)	185 (62.3 %	
Total	48 (100 %)	1307 (100 %)	953 (100 %)	297 (100 %)

## Discussion

Results from the present study indicate a high level of concordance between self-reported pre-pregnancy BMI and BMI measured at the first prenatal study contact (mean gestational age = 10.9 ± 3.3 weeks). Self-reported weight was significantly lower than measured weight; and, self-reported height was significantly higher than measured height. This resulted in significantly higher measured BMI than pre-pregnancy BMI obtained from self-report.

The observed mean difference between self-reported pre-pregnancy weight and measured weight (0.27 kg) was smaller when compared to similar values reported in studies among women living in high-income countries. For instance, among US women, Shin et al. using data from the National Health and Nutrition Examination Survey found that self-reported pre-pregnancy weight was 2.3 kg lower compared with weight measured in the first trimester of pregnancy [19]. Similarly Holland and colleagues in their study of pregnant women receiving prenatal care at obstetric clinics in Massachusetts reported that self-reported pre-pregnancy weight was 1.8 kg lighter than weight measured at the first prenatal visit (mean gestational age =9.7 ± 2.1 weeks) [20]. The observation of a small mean difference between self-reported pre-pregnancy and measured weight in the present study potentially suggests that women in this study were more truthful when recalling their most recent measured weight. It is possible that women recalled weight measured in the index pregnancy, in their homes or at the INMP clinic or at other clinics, but before enrollment into the present study. Women in this study may have weighed themselves in their homes and in the index pregnancy but prior to when we enrolled them. It is, however, unlikely that women in our study had reported to the INMP or other clinics in the index pregnancy at gestational ages that were earlier than the 10.9 ± 3.3 weeks of gestation (the gestational age at which women were enrolled into the present study). The observed differences between the self-reported pre-pregnancy and measured weights, therefore, may be due to other reasons. These include, for example, that women in this study experienced minimal weight gain following conception to the time of their first prenatal study visit and weight measurement. Physiological conditions of pregnancy such as nausea, vomiting and hyperemesis gravidarum might have acted separately or jointly to minimize weight gained prior to recruitment into the PrOMIS study cohort. There might also have been small inconsistencies in the recalled and actual measurements such as differences in the scales used, clothing, time of day and voiding status. As such women’s recalled pre-pregnancy weight was very close or similar to values measured at the first contact due to a combination of factors.

A large mean difference between self-reported and measured height (2 cm) was observed in this study. Limited studies have documented differences between self-reported and measured height among pregnant women living in higher income countries or LMICs. For example, Paez et al. studied 30 pregnant women participating in an intervention to prevent postpartum diabetes and reported a 0.56 cm higher? difference in self-reported and measured height during pregnancy [21]. Studies involving non-pregnant women have also reported relatively lower mean differences between self-reported and measured height e.g. 0.70 cm among a healthy overweight working women in the Netherlands [22] or 0.30 cm among female general practice patients in Australia. It is possible that the large difference between measured and self-reported height observed this study is largely due to social desirability bias i.e. women preferring to mention that they are taller than they are actually are or due to other unknown factors that need to be examined in future studies e.g. through formative studies.

We did not have measured pre-pregnancy weight and height; and, this study does not fully address the question of how reliable or valid pre-pregnancy BMI is compared to measured pre-pregnancy BMI. Behavioral and economic reasons hinder access to pre-conceptional care upon which pre-pregnancy BMI can be measured [29]. We observed small differences between self-reported pre-pregnancy BMI and BMI obtained at the first prenatal visit in this study; and, assuming minimal weight gain during the early pregnancy period, it is reasonable to recommend use of self-reported pre-pregnancy BMI or BMI obtained from values measured early during pregnancy as proxies for measured pre-pregnancy BMI. Using either or both of these BMI proxies would be particularly helpful for providing appropriate weight gain counseling during the perinatal period.

Our study has several strengths. This study used a large sample size and is the first study to examine differences in self-reported pre-pregnancy BMI and BMI obtained early during pregnancy among women living in a non-high income country. However, there are some limitations to our findings. Most women in this study (95.1 %) had seven or more years of education. Our results may not be generalizable in LMICs contexts where education levels among pregnant women in antenatal care may be lower. Also, were not able to establish true reliability and validity of self-reported pre-pregnancy BMI as we did not have measured pre-pregnancy weight or height. Lastly, a substantial proportion of pregnant women in this study (17.6 %) did not provide any self-reported pre-pregnancy weight or height possibly indicating that they did not know these measures. Investigators planning to use self-reported pre-pregnancy weight or height among Peruvian pregnant women or women living in other similar contexts need to be aware that a substantial proportion of their study participants may not know such measures. Inability to recall pre-pregnancy anthropometrics probably lends further support to using weight and height measured early during the pregnancy to calculate early pregnancy BMI as a proxy for pre-pregnancy nutritional status.

## Conclusions

The BMI obtained using self-reported pre-pregnancy weight and height strongly correlated with BMI obtained using anthropometrics measured at the first prenatal study contact. This potentially suggests that, in this context, there is minimal change between pre-pregnancy weight and similar anthropometrics measured early during pregnancy; or, that women in this study just recalled their most recently measured height and weight (including values obtained during the index pregnancy but before the first prenatal study visit). In this or similar contexts, we recommend use of weight and height measured early during pregnancy; and, then calculating the early pregnancy BMI as a proxy for pre-pregnancy BMI.

## Additional file

Additional file 1: Table S1. Socio-demographic and lifestyle characteristics of participants in the PrOMIS cohort by whether they provided self-reported body mass index (BMI) or not. (DOCX 14 kb)
