# Supplementary material for: Concordance between self-reported pre-pregnancy body mass index (BMI) and BMI measured at the first prenatal study contact
Source: BMC Pregnancy Childbirth. 2016 Jul 26;16:187. doi: 10.1186/s12884-016-0983-z (PMC4962409; doi:10.1186/s12884-016-0983-z)
Supplement: Additional file 1: Table S1. — Socio-demographic and lifestyle characteristics of participants in the PrOMIS cohort by whether they provided self-reported body mass index (BMI) or not. (DOCX 14 kb) [file 12884_2016_983_MOESM1_ESM.docx]

**Supplementary table 1.** Socio-demographic and lifestyle characteristics of participants in the PrOMIS cohort by whether they provided self-reported body mass index (BMI) or not

|  | **Self-reported pre-pregnancy BMI* available?** | |  |
| --- | --- | --- | --- |
| **Variable** | **Yes (Mean ±SD** or (%))** | **No (Mean ±SD** or (%))** | **P value** |
| Gestational age at recruitment | 9.4±3.3 | 9.1±3.3 | 0.039 |
| Maternal age | 28.0±6.2 | 29.0±6.6 | 0.001 |
| Of Mestizo ethnicity (Mixed race ancestry) | 76.2% | 71.5% | 0.024 |
| Married or living with partner | 80.7% | 84.1% | 0.072 |
| Nulliparous | 50.7% | 40.8% | <0.001 |
| Seven or more years of education | 96.1% | 93.9% | 0.022 |
| Employed | 46.1% | 48.0% | 0.429 |
| Planned pregnancy | 42.2% | 41.5% | 0.774 |

*BMI: Body Mass Index; **SD: Standard Deviation
